# Neurocognitive and Motor Performance in Pediatric Kidney Transplant Recipients: A Cross-Sectional Controlled Study

**DOI:** 10.3390/children13091145

**Published:** 2026-08-26

**Authors:** Seyma Tirpanci, Meltem Kaya, Hikmet Ucgun, Mehmet Tasdemir, Hilal Denizoglu Kulli, Ozan Ozkaya

**Affiliations:** 1 Physiotherapy and Rehabilitation Master’s Program, Institute of Graduate Education, Atlas University, Istanbul 34403, Türkiye; symoznn@gmail.com; 2 Department of Physiotherapy and Rehabilitation, Faculty of Health Sciences, Atlas University, Istanbul 34403, Türkiye; hikmet.ucgun@atlas.edu.tr (H.U.); hilal.kulli@atlas.edu.tr (H.D.K.); 3 Department of Pediatric Nephrology, Faculty of Medicine, Istinye University, Istanbul 34475, Türkiye; mehmet.tasdemir@istinye.edu.tr (M.T.); ozan.ozkaya@istinye.edu.tr (O.O.)

**Keywords:** balance, child, cognition, kidney transplantation, motor skills

## Abstract

**Highlights:**

**What are the main findings?**

• Pediatric kidney transplant recipients showed poorer executive function and manual dexterity than age- and sex-matched healthy peers.
• Dominant-hand and cognitive-task reaction times were prolonged, whereas handgrip strength and functional balance were relatively preserved.

**What are the implications of the main findings?**

• Long-term post-transplant follow-up should include standardized cognitive and motor assessments in addition to routine monitoring of renal and graft function.
• Early identification of residual impairments may support individualized rehabilitation targeting executive function, manual dexterity, and cognitive–motor integration.

**Abstract:**

**Background/Objectives**: Although kidney transplantation improves survival and quality of life in children with end-stage kidney disease, neurocognitive and motor impairments acquired during chronic kidney disease (CKD) may persist after transplantation. Cognitive–motor evaluations in this population remain limited. This study compared cognitive function, manual dexterity, grip strength, reaction time, and balance between pediatric kidney transplant recipients and healthy peers. **Methods**: This study included 30 pediatric kidney transplant recipients aged 8–18 years and 30 age- and sex-matched healthy controls. Cognitive function was assessed using the Stroop Color–Word Test. Fine and gross manual dexterity were evaluated using the Nine-Hole Peg Test and Box and Block Test, respectively. Handgrip strength was measured with a JAMAR dynamometer, reaction time with the FitLight Trainer™ system, and balance with the Pediatric Balance Scale. **Results**: Compared with healthy controls, pediatric kidney transplant recipients showed poorer executive function, demonstrated by longer Stroop completion times and more color–word errors (all *p* < 0.05). Manual dexterity was reduced, with a significantly lower Box and Block Test score for the dominant hand (*p* = 0.020) and longer Nine-Hole Peg Test completion times for both hands (both *p* < 0.001), whereas the between-group difference in the non-dominant Box and Block Test did not reach statistical significance (*p* = 0.055). Dominant-hand reaction time and reaction time during the cognitive task were significantly prolonged (*p* = 0.018 and *p* = 0.046, respectively). Handgrip strength and Pediatric Balance Scale scores did not differ significantly between groups (both *p* > 0.05). **Conclusions**: Pediatric kidney transplant recipients exhibit persistent impairments in executive function, manual dexterity, and cognitive–motor performance despite transplantation. Long-term follow-up should incorporate cognitive and motor assessments to support early rehabilitation and optimize functional outcomes.

## 1. Introduction

Chronic kidney disease (CKD) in childhood is a lifelong condition that extends far beyond progressive loss of renal function, affecting physical growth, neurodevelopment, cardiovascular health, educational achievement, and overall quality of life [1]. Although pediatric CKD is considerably less common than adult CKD, its onset during critical periods of brain maturation may adversely influence lifelong cognitive, physical, and psychosocial development [2]. Congenital anomalies of the kidney and urinary tract remain the leading cause of pediatric CKD worldwide, and a substantial proportion of these patients eventually progress to end-stage kidney disease (ESKD), requiring kidney replacement therapy [3]. Improvements in pediatric nephrology, surgical techniques, and immunosuppressive therapy have markedly increased patient and graft survival over the past two decades, shifting the primary focus of care from survival alone toward preserving long-term cognitive, physical, and psychosocial functioning [1,4].

Pediatric kidney transplantation is widely recognized as the treatment of choice for children with ESKD because it offers superior patient survival, graft longevity, growth potential, and health-related quality of life compared with long-term dialysis [5]. Restoration of kidney function following transplantation corrects many metabolic disturbances associated with CKD, enabling improved nutritional status, school attendance, and social participation [1]. Nevertheless, transplantation does not fully reverse the cumulative effects of prolonged uremia, chronic inflammation, hypertension, anemia, dialysis exposure, or disease-related disruptions that occur during critical stages of neurodevelopment [2,6]. Furthermore, long-term immunosuppressive therapy, recurrent hospitalizations, and persistent metabolic or cardiovascular complications may continue to influence central nervous system maturation and physical performance despite successful transplantation [1,7]. Consequently, increasing attention has shifted from traditional transplant outcomes, such as patient and graft survival, toward the evaluation of functional outcomes that better reflect children’s long-term health, independence, and participation in everyday life [5,8].

Because CKD develops during a critical period of brain maturation, the developing brain is particularly susceptible to systemic metabolic disturbances associated with ESKD [2,6]. Chronic exposure to uremic toxins, persistent inflammation, hypertension, anemia, oxidative stress, and endothelial dysfunction may adversely affect cerebral perfusion, white matter integrity, and neural connectivity during critical stages of neurodevelopment [2,9]. Although transplantation improves renal physiology, evidence suggests that structural and functional alterations acquired before transplantation may persist, resulting in long-term impairments in attention, executive functioning, processing speed, and working memory [1,8]. These neurocognitive deficits may also influence motor performance because skilled motor performance relies on the coordinated interaction between executive function, sensory integration, and neuromuscular control [8,9]. Consequently, deficits in hand dexterity, reaction time, muscle performance, and postural control may continue to compromise daily activities, academic achievement, and social participation despite successful transplantation [1,10].

Most previous studies have primarily focused on global cognitive outcomes or health-related quality of life, whereas motor performance has generally been evaluated using isolated measures such as muscle strength or physical fitness [1,7,9]. Comprehensive assessments integrating neurocognitive and motor outcomes in pediatric kidney transplant recipients remain scarce [8,9]. In particular, studies simultaneously evaluating upper-extremity dexterity, grip strength, reaction time, and postural balance alongside cognitive performance are lacking [10]. Therefore, the present study aimed to assess cognitive function, hand dexterity, grip strength, reaction time, and balance in children who had undergone kidney transplantation and to compare these outcomes with those of healthy peers.

## 2. Materials and Methods

### 2.1. Study Design and Participants

This cross-sectional controlled study was conducted at the Department of Pediatric Nephrology, Istinye University, and the Department of Physiotherapy and Rehabilitation, Istanbul Atlas University. The study included pediatric kidney transplant recipients who were routinely followed at the pediatric nephrology outpatient clinic and age- and sex-matched healthy controls recruited from the local community between May 2024 and March 2025.

Thirty children who had undergone kidney transplantation and thirty healthy peers were enrolled in the study. Eligible participants in the transplant group were between 8 and 18 years of age, had received a kidney transplant at least six months before enrollment, and were clinically stable without acute medical complications at the time of assessment. Healthy controls were recruited to match the transplant group with respect to age and sex and had no history of renal, neurological, orthopedic, cardiovascular, or developmental disorders. Participants in both groups were excluded if they had any neurological disorder, developmental delay, musculoskeletal disease affecting upper-extremity function or balance, generalized joint hypermobility, previous upper-extremity surgery, or any condition that could interfere with cognitive or motor performance assessments.

The study protocol was approved by the Ethics Committee of Istanbul Atlas University (Approval Number: E-22686390-050.99-46516; Date: 22 July 2024) and was conducted in accordance with the principles of the Declaration of Helsinki. Written informed consent was obtained from the parents or legal guardians of all participants, and assent was obtained from children whenever appropriate.

### 2.2. Outcome Measures

All assessments were performed by the same experienced physiotherapist during a single testing session. Participants were instructed and familiarized with each assessment before testing. Standardized verbal instructions and testing procedures were used throughout the study. To minimize the potential effects of fatigue, assessments were performed in the following order: cognitive function, hand dexterity, grip strength, reaction time, and balance. Standardized rest periods were provided between consecutive tests.

### 2.3. Demographic Characteristics

Demographic characteristics, including age, sex, height, body weight, and body mass index (BMI), were recorded for all participants. In the kidney transplant group, additional clinical information was collected from medical records, including primary kidney disease, history and duration of dialysis, time since transplantation, donor type, current immunosuppressive medications, biochemical parameters, and accompanying comorbidities.

### 2.4. Cognitive Function

Cognitive function was assessed using the Stroop Color–Word Test, a widely used neuropsychological instrument that evaluates selective attention, inhibitory control, cognitive flexibility, and processing speed [11,12]. Participants completed the standardized four-part test protocol. Completion time and the number of errors for each subtest were recorded. Longer completion times and higher error rates indicated poorer cognitive performance.

### 2.5. Hand Dexterity

Fine manual dexterity was evaluated using the Nine-Hole Peg Test (NHPT) [13], while gross manual dexterity was assessed using the Box and Block Test (BBT) [14]. Both dominant and non-dominant hands were evaluated according to standardized procedures. For the NHPT, the time required to place and remove nine pegs was recorded in seconds, with shorter completion times indicating better dexterity. For the BBT, the number of blocks transferred from one compartment to another within 60 s was recorded, with higher scores reflecting better gross manual dexterity.

### 2.6. Grip Strength

Handgrip strength was measured using a hydraulic grip dynamometer (JAMAR^®^, Patterson Medical, Warrenville, IL, USA) with the patient seated on a chair, the shoulder abducted and neutrally rotated, the elbow flexed at 90°, and the forearm and wrist in a neutral position [15]. The maximum value of three consecutive measurements taken from the dominant side was recorded. Children were given approximately 1 min of rest between efforts.

### 2.7. Reaction Time

Reaction time was assessed using the FitLight Trainer™ System (FITLIGHT^®^ Corp., Miami, FL, USA), an interactive visual reaction training and assessment device [16]. Participants were instructed to respond as quickly as possible to randomly illuminated targets using both dominant and non-dominant hands. Mean, fastest, and slowest reaction times were recorded for each hand. Longer reaction times represented poorer visuomotor performance.

### 2.8. Balance

Balance was evaluated using the Pediatric Balance Scale (PBS), a functional balance assessment consisting of 14 tasks performed under standardized conditions [17]. Each item is scored on a five-point ordinal scale ranging from 0 to 4, yielding a maximum total score of 56. Higher scores indicate better functional balance.

### 2.9. Statistical Analysis and Sample Size

Statistical analysis was conducted using IBM SPSS v.26 (IBM Corp., Armonk, NY, USA). The normality of the distribution of data was analyzed using the Shapiro–Wilk Test. Continuous variables are presented as mean ± standard deviation (SD) for normally distributed data and median for non-normally distributed data, whereas categorical variables are expressed as frequency (n) and percentage (%). Between-group comparisons were performed using the independent-samples *t*-test for normally distributed continuous variables and the Mann–Whitney U test for non-normally distributed variables. Categorical variables were compared using the chi-square test or Fisher’s exact test, when appropriate. A one-sample *t*-test was used to compare handgrip strength values in the kidney transplantation group with published age- and sex-specific normative reference values. All statistical tests were two-tailed, and a *p* < 0.05 was considered statistically significant.

The G*Power 3.1 (University of Düsseldorf, Düsseldorf, Germany) software was used for the sample size calculation [18]. The calculation was based on a previously reported difference in handgrip strength between children with chronic kidney disease receiving dialysis and healthy controls, corresponding to a Cohen’s d of 0.751 [19]. Assuming a two-sided significance level (α) of 0.05 and a statistical power of 80%, a minimum sample size of 29 participants per group was required. Therefore, 30 participants were enrolled in each group.

## 3. Results

A total of 60 participants, including 30 pediatric kidney transplant recipients and 30 age- and sex-matched healthy controls, completed all assessments. The demographic and clinical characteristics of the participants are presented in Table 1. There were no significant differences between the kidney transplantation and control groups regarding age, sex distribution, height, weight, body mass index, or dominant side (all *p* > 0.05. Clinical characteristics specific to the kidney transplantation group, including disease duration, post-transplant duration, dialysis history, and tacrolimus dosage, are also summarized in Table 1.

Compared with healthy controls, pediatric kidney transplant recipients demonstrated significantly poorer cognitive performance, requiring longer completion times on the Stroop Word Reading (27.88 ± 10.47 vs. 21.24 ± 3.27 s, *p* < 0.001), Color Naming (29.02 ± 11.22 vs. 23.49 ± 5.34 s, *p* = 0.002), and Color–Word Reading (37.45 ± 9.59 vs. 30.82 ± 6.31 s, *p* = 0.007) tasks, and making more Color–Word errors (3.92 ± 3.46 vs. 1.32 ± 1.58, *p* = 0.001). The kidney transplantation group also exhibited lower manual dexterity. Box and Block Test scores were significantly lower for the dominant hand (54.21 ± 9.60 vs. 59.90 ± 8.36, *p* = 0.020), whereas the difference for the non-dominant hand did not reach statistical significance (53.17 ± 8.37 vs. 57.40 ± 8.37, *p* = 0.055). In contrast, Nine-Hole Peg Test completion times were significantly longer in the kidney transplantation group for both the dominant (26.40 ± 6.01 vs. 21.74 ± 5.39 s, *p* < 0.001) and non-dominant hands (27.13 ± 3.44 vs. 24.50 ± 5.38 s, *p* < 0.001). The kidney transplantation group also demonstrated a significantly longer dominant-hand reaction time (0.39 ± 0.22 vs. 0.29 ± 0.10 s, *p* = 0.018) and cognitive reaction time (0.58 ± 0.14 vs. 0.51 ± 0.10 s, *p* = 0.046) than the control group. No significant between-group differences were observed in handgrip strength, non-dominant or bilateral reaction time, or Pediatric Balance Scale scores (all *p* > 0.05) (Table 2).

## 4. Discussion

This study aimed to compare neurocognitive and motor performance between pediatric kidney transplant recipients and healthy peers. To our knowledge, this is the first case–control study to comprehensively evaluate executive function, manual dexterity, reaction time, handgrip strength, and balance in pediatric kidney transplant recipients. The principal findings were that pediatric kidney transplant recipients demonstrated impaired executive function, reduced fine manual dexterity bilaterally, reduced gross manual dexterity in the dominant hand, and prolonged dominant-hand and cognitive reaction times compared with healthy controls. In contrast, handgrip strength and balance performance did not differ significantly between the groups.

Despite successful kidney transplantation, neurocognitive deficits frequently persist in pediatric recipients, suggesting that restoration of kidney function alone is insufficient to reverse CKD-related brain injury completely [1,6]. The developing brain is particularly vulnerable to prolonged exposure to uremic toxins, chronic inflammation, hypertension, anemia, oxidative stress, endothelial dysfunction, and dialysis-related metabolic disturbances, which may impair cerebral perfusion, delay white matter maturation, and disrupt frontostriatal and frontoparietal networks, thereby compromising executive functioning, attention, and processing speed [20,21]. Furthermore, longer dialysis exposure before transplantation and the potential neurotoxic effects of long-term calcineurin inhibitor therapy may further contribute to persistent executive dysfunction after transplantation [1]. Consistent with these mechanisms, pediatric kidney transplant recipients in our study demonstrated significantly poorer performance across all Stroop subtests, including word reading, color naming, color–word interference, and error scores, indicating persistent impairments in processing speed, selective attention, inhibitory control, and executive functioning compared with healthy controls. The sensitivity of the Stroop Color–Word Test to subtle executive dysfunction suggests persistent impairment of higher-order executive networks, particularly those governing inhibitory control, cognitive flexibility, and processing speed. This interpretation is supported by accumulating evidence demonstrating that executive function is one of the cognitive domains most consistently affected across the pediatric CKD continuum. Although kidney transplantation substantially improves metabolic status, impairments in attention regulation, working memory, processing speed, and executive functioning frequently persist because neurodevelopmental injury begins before transplantation and may only partially recover thereafter [6,22,23,24]. Collectively, these findings indicate that successful kidney transplantation may restore renal function while failing to fully normalize higher-order executive processes.

Motor impairments may persist after pediatric kidney transplantation, indicating that restoration of renal function does not necessarily normalize sensorimotor development [25]. Manual dexterity relies on the integration of motor control, visuomotor processing, attention, and motor planning. Consequently, neurological injury acquired during CKD, together with post-transplant immunosuppression, may lead to persistent deficits in sensorimotor integration and coordinated upper-limb movements [26]. Consistent with these mechanisms, pediatric kidney transplant recipients in our study demonstrated significantly poorer performance on the Nine-Hole Peg Test for both the dominant and non-dominant hands, indicating bilateral impairment in fine manual dexterity. For gross manual dexterity, Box and Block Test performance was significantly lower for the dominant hand, whereas the lower score observed for the non-dominant hand did not reach statistical significance. This pattern suggests that fine manual dexterity impairment may be more consistently expressed bilaterally, whereas deficits in gross manual dexterity may be more selective. These findings are consistent with previous studies reporting persistent deficits in motor coordination, manual performance, and visuomotor integration after pediatric kidney transplantation, suggesting that motor dysfunction may originate during the CKD period and may continue despite successful transplantation [1,27]. In addition, calcineurin inhibitors, particularly tacrolimus, are well recognized to be associated with neurological adverse effects, including tremor, impaired coordination, and peripheral neuropathy, which may further compromise manual dexterity, although their independent contribution remains uncertain [28]. Although these associations have been reported in adult kidney transplant recipients, impaired manual dexterity during childhood may similarly interfere with handwriting, self-care, school activities, and participation in age-appropriate physical and social activities. Taken together, our findings indicate that bilateral deficits in fine manual dexterity and more selective impairments in gross manual dexterity may persist following pediatric kidney transplantation, supporting routine motor assessment and early rehabilitation as part of long-term post-transplant care.

Hand grip strength is a simple and reliable indicator of overall muscle strength, physical fitness, and neuromuscular function in children, reflecting the combined influence of muscle mass, nutritional status, physical activity, and neuromuscular integrity [29]. Chronic kidney disease may adversely affect muscle development through chronic inflammation, metabolic disturbances, reduced physical activity, malnutrition, growth impairment, and prolonged dialysis exposure, with some deficits persisting despite successful kidney transplantation [30]. Although pediatric kidney transplant recipients exhibited lower mean hand grip strength than healthy controls, the observed between-group differences did not achieve statistical significance. However, comparison with published age- and sex-specific normative values using a one-sample *t*-test demonstrated significantly lower grip strength in the transplant group, indicating that muscle strength remained below the expected level for healthy children despite the absence of a significant between-group difference [31,32]. The lack of statistical significance in the comparison with our control group may reflect the limited sample size and heterogeneity in factors such as age, pubertal status, physical activity, nutritional status, and time since transplantation. Although the between-group difference was not statistically significant in the present study, previous studies have reported reduced hand grip strength and peripheral muscle strength in pediatric kidney transplant recipients compared with healthy peers [33,34]. Frantzeski et al. suggested that persistent muscle weakness following pediatric kidney transplantation may reflect incomplete recovery from CKD-related muscle abnormalities, while Barbosa et al. speculated that chronic inflammation, impaired growth, reduced physical activity, and CKD-related alterations in muscle metabolism may contribute to impaired muscle strength in this population [33,34]. Overall, our findings suggest that lower hand grip strength relative to published normative values may indicate subtle residual impairment, supporting routine monitoring during long-term follow-up after pediatric kidney transplantation.

Reaction time is a sensitive indicator of cognitive–motor integration that depends on the coordinated interaction of sensory processing, selective attention, response selection, and motor execution, rather than reflecting motor performance alone [35]. In pediatric CKD, subtle deficits in attention, executive functioning, and processing speed have been documented, and these abnormalities may persist after transplantation because pre-transplant neurodevelopmental injury is only partially reversible [1]. Because of this incomplete recovery, persistent deficits in cognitive–motor performance may still be observed in pediatric kidney transplant recipients. In our study, pediatric kidney transplant recipients demonstrated significantly prolonged dominant-hand and reaction time during the cognitive task compared with healthy controls, whereas non-dominant and bilateral measures did not differ significantly. This selective pattern suggests that reaction-time impairment may not represent a generalized slowing of all motor responses, but rather reduced efficiency in attentional processing, visuomotor integration, and response preparation under specific task conditions. The coexistence of prolonged reaction times, impaired Stroop performance, and reduced manual dexterity in our cohort further suggests that these abnormalities may share common mechanisms involving disrupted cognitive–motor integration rather than representing isolated deficits in individual functional domains. Previous reaction-time studies in patients with ESKD have suggested that slowed responses primarily reflect deficits in attentional control and response preparation rather than isolated motor impairment [36]. These observations support the possibility that the prolonged reaction times observed in our cohort reflect persistent CKD-related alterations in cognitive–motor processing rather than reduced motor speed alone, despite successful transplantation. Furthermore, studies in pediatric CKD have demonstrated impairments in focused and sustained attention, while reviews of pediatric kidney transplantation suggest that processing speed and executive function remain vulnerable despite restoration of renal function [22,37]. Reaction time is also closely related to manual performance because speeded motor responses depend on intact visuomotor processing and motor planning. Studies in children have demonstrated associations between slower reaction time, poorer fine motor performance, and reduced visuomotor integration [38]. Accordingly, the parallel prolongation of reaction time and impairment in performance on the Nine-Hole Peg Test and Box and Block Test observed in our cohort may indicate a broader disturbance in cognitive–motor processing. Overall, these findings suggest that reaction time may represent a sensitive marker of persistent cognitive–motor dysfunction after pediatric kidney transplantation and support its inclusion in long-term functional assessment alongside conventional cognitive and motor evaluations.

Balance is a complex motor function that depends on the integration of vestibular, visual, and somatosensory inputs with adequate muscle strength, proprioception, and motor coordination, making it an important indicator of neuromuscular function [39]. In the present study, PBS scores were lower in pediatric kidney transplant recipients than in healthy controls; however, the difference did not reach statistical significance. Although these findings do not indicate overt balance impairment at the group level, the lower mean score observed in the transplant group may suggest subtle deficits in postural control. This interpretation should be approached with caution because the PBS is known to exhibit ceiling effects in healthy children, potentially limiting its ability to detect mild balance impairments in relatively high-functioning individuals. Consequently, small differences in postural stability may not be adequately captured despite clinically relevant individual variability. Persistent neuromuscular alterations following CKD and kidney transplantation, including reduced muscle strength, prolonged physical inactivity, impaired sensorimotor function, and the potential neurological effects of immunosuppressive therapy, may contribute to residual impairments in postural control [40]. Although studies evaluating balance in pediatric kidney transplant recipients remain limited, investigations in adult transplant recipients have demonstrated impaired static and dynamic balance, particularly under more challenging sensory and dual-task conditions, suggesting that subtle postural control deficits may persist despite successful transplantation [41,42]. The absence of a significant between-group difference in our study may therefore reflect the limited sensitivity of the PBS rather than the complete preservation of balance function. More sensitive, instrumented balance assessments, such as computerized posturography, may better identify subtle postural abnormalities that are not detected by clinical balance scales. Overall, our findings suggest that while gross balance performance appears largely preserved after pediatric kidney transplantation, subtle impairments in postural control may still be present and warrant further evaluation using more sensitive assessment methods during long-term follow-up.

This study has several limitations. First, the sample size was not sufficient to support multivariable regression or meaningful subgroup analyses. Consequently, potential sources of clinical heterogeneity, including primary kidney disease, pre-transplant dialysis exposure and duration, and time since transplantation, could not be examined in relation to neurocognitive and motor outcomes and may represent residual confounding. Furthermore, the cross-sectional design precludes causal inference and does not allow for assessment of longitudinal trajectories of recovery or persistent neurodevelopmental impairment following kidney transplantation. Second, balance was assessed only using the Pediatric Balance Scale, which may have been insufficiently sensitive to detect subtle postural control deficits because of its known ceiling effects. Finally, physical activity levels were not evaluated, although they may influence motor and cognitive performance in pediatric kidney transplant recipients. Future longitudinal studies with larger cohorts are needed to enable adequately powered multivariable and subgroup analyses and to better characterize the influence of clinical heterogeneity and post-transplant recovery over time. More sensitive, instrumented balance assessments and comprehensive evaluation of physical activity should also be considered.

## 5. Conclusions

In conclusion, pediatric kidney transplant recipients demonstrated persistent impairments in several domains of cognitive and motor function despite successful transplantation. Executive function, reaction time, and manual dexterity were significantly affected compared with healthy peers, whereas hand grip strength and balance appeared to be relatively preserved, although subtle functional deficits may still be present. These findings suggest that restoration of renal function alone may not completely reverse the neurodevelopmental and neuromuscular consequences of chronic kidney disease acquired during childhood. Therefore, comprehensive long-term follow-up should extend beyond routine graft monitoring to include standardized assessments of cognitive and motor function. Early identification of residual impairments may facilitate timely rehabilitation and individualized interventions aimed at improving functional outcomes and quality of life in pediatric kidney transplant recipients.

## Figures and Tables

**Table 1:** The demographic and clinical characteristics of all participants.

	Kidney Transplantation Group (*n* = 30)	Control Group (*n* = 30)	*p*
**A** **ge (years)**	14.11 ± 3.41	13.37 ± 2.69	0.332
**Gender**			
Female (*n*; %)	18 (60%)	16 (53.3%)	0.602
Male (*n*; %)	12 (40%)	14 (46.7%)	
**Body composition**			
Height (cm)	147.19 ± 14.89	154.23 ± 13.30	0.125
Weight (kg)	47.83 ± 16.06	51.93 ± 14.78	0.145
BMI (kg/m^2^)	21.62 ± 5.21	20.30 ± 3.79	0.554
**Dominant side**			
Right	27 (90%)	28 (93.4%)	0.055
Left	3 (10%)	2 (6.6%)	
**Biochemical parameters**			
BUN (mg/dL)	17.20 ± 6.07	-	-
Creatinine (mg/dL)	0.91 ± 0.30	-	-
eGFR (mL/min/1.73 m^2^)	72.00 ± 22.62	-	-
Sodium (mmol/L)	142.73 ± 12.90	-	-
Potassium (mmol/L)	4.28 ± 0.59	-	-
Calcium (mg/dL)	15.57 ± 2.81	-	-
Phosphorus (mg/dL)	7.73 ± 1.46	-	-
Magnesium (mg/dL)	1.68 ± 0.16	-	-
**Primary kidney disease**			
Nephronophthisis	10 (33.3%)		
Nephrotic syndrome	6 (20%)		
Cystinosis	6 (20%)		
Neurogenic bladder	2 (6.7%)		
Hypoplastic kidney	2 (6.7%)		
VATER syndrome	2 (6.7%)		
CAKUT/VUR	2 (6.7%)		
**Duration of chronic kidney disease (months)**	88.95 ± 79.19	-	-
**Time since kidney transplantation (months)**	30.96 ± 22.89	-	-
**Dialysis status**			
Hemodialysis (*n*; %)	3 (10%)	-	-
Peritoneal dialysis (*n*; %)	9 (30%)		
Hemodialysis + Peritoneal dialysis (*n*; %)	5 (16.7%)		
**Duration of dialysis (months)**	20.20 ± 28.17	-	-
**Tacrolimus daily dose (mg/kg/day)**	0.09 ± 0.06	-	-

Data are presented as mean ± standard deviation or n (%). BMI: body mass index; BUN: blood urea nitrogen; CAKUT, congenital anomalies of the kidney and urinary tract; VUR, vesicoureteral reflux; VATER, vertebral defects, anal atresia, tracheoesophageal fistula, esophageal atresia, and renal anomalies. cm: centimeter; kg: kilogram.

**Table 2:** Comparison of outcome measures between the kidney transplantation and control groups.

	Kidney Transplantation Group (n = 30)	Control Group (n = 30)	*p* Value	95% CI for Mean Difference		Effect Size (Cohen’s *d*)
				Lower	Upper	
**Stroop Test**						
Word Reading Time (s)	27.88 ± 10.47	21.24 ± 3.27	<0.001 *	2.63	10.65	0.85
Color Naming Time (s)	29.02 ± 11.22	23.49 ± 5.34	0.002 *	0.99	10.07	0.62
Color–Word Reading Time (s)	37.45 ± 9.59	30.82 ± 6.31	0.007 *	2.44	10.82	0.81
Color–Word Errors (n)	3.92 ± 3.46	1.32 ± 1.58	0.001 *	1.21	3.99	0.98
**Box and Block Test**						
Dominant (n)	54.21 ± 9.60	59.90 ± 8.36	0.020 *	−10.34	−1.04	0.63
Non-dominant (n)	53.17 ± 8.37	57.40 ± 8.37	0.055	−8.56	0.10	0.51
**9-Hole Peg Test**						
Dominant (s)	26.40 ± 6.01	21.74 ± 5.39	<0.001 *	1.71	7.61	0.82
Non-dominant (s)	27.13 ± 3.44	24.50 ± 5.38	<0.001 *	0.30	4.96	0.58
**Handgrip Strength**						
Dominant (kg)	20.13 ± 8.08	23.53 ± 6.91	0.336	−7.28	0.48	0.45
Non-dominant (kg)	18.87 ± 7.04	20.38 ± 6.53	0.348	−5.02	2.00	0.22
**Upper Extremity Reaction Time**						
Mean reaction time (dominant hand) (s)	0.39 ± 0.22	0.29 ± 0.10	0.018 *	0.10	0.19	0.58
Mean reaction time (non-dominant hand) (s)	0.32 ± 0.10	0.28 ± 0.06	0.171	−0.004	0.085	0.47
Mean reaction time (bilateral hand) (s)	0.28 ± 0.09	0.29 ± 0.08	0.320	−0.054	0.034	0.12
Mean reaction time (under cognitive task) (s)	0.58 ± 0.14	0.51 ± 0.10	0.046 *	0.007	0.133	0.58
**Balance**						
Pediatric Balance Scale	50.97 ± 13.96	54.57 ± 2.20	0.276	−8.76	1.56	0.36

Data are presented as mean ± SD. Between-group differences are reported with 95% confidence intervals (CIs). * *p* ≤ 0.05.

## Data Availability

The data presented in this study are available from the corresponding author upon reasonable request. The data are not publicly available due to privacy and ethical considerations related to the study participants.

## Abbreviations

BBT	Box and Block Test
BMI	Body mass index
BUN	Blood urea nitrogen
CAKUT	Congenital anomalies of the kidney and urinary tract
CI	Confidence interval
CKD	Chronic kidney disease
ESKD	End-stage kidney disease
NHPT	Nine-Hole Peg Test
PBS	Pediatric Balance Scale
VATER	Vertebral anomalies, anal atresia, tracheoesophageal fistula with esophageal atresia, and renal anomalies.
VUR	Vesicoureteral reflux
